# *Candida* Infections and Therapeutic Strategies: Mechanisms of Action for Traditional and Alternative Agents

**DOI:** 10.3389/fmicb.2018.01351

**Published:** 2018-07-03

**Authors:** Giselle C. de Oliveira Santos, Cleydlenne C. Vasconcelos, Alberto J. O. Lopes, Maria do S. de Sousa Cartágenes, Allan K. D. B. Filho, Flávia R. F. do Nascimento, Ricardo M. Ramos, Emygdia R. R. B. Pires, Marcelo S. de Andrade, Flaviane M. G. Rocha, Cristina de Andrade Monteiro

**Affiliations:** ^1^Programa de Doutorado em Biotecnologia da Rede Nordeste de Biotecnologia (RENORBIO), Universidade Federal do Maranhão, São Luís, Brazil; ^2^Postgraduate Program in Health Sciences, Universidade Federal do Maranhão, São Luís, Brazil; ^3^Departamento de Engenharia Elétrica, Programa de Doutorado em Biotecnologia da Rede Nordeste de Biotecnologia (RENORBIO), Universidade Federal do Maranhão, São Luís, Brazil; ^4^Departamento de Patologia, Universidade Federal do Maranhão, São Luís, Brazil; ^5^Department of Information, Environment, Health and Food Production, Laboratory of Information Systems, Federal Institute of Piauí, Teresina, Brazil; ^6^Department of Biology, Federal University of Maranhão, São Luís, Brazil; ^7^Laboratório de Micologia Médica, Programa de Mestrado em Biologia Parasitária, Universidade Ceuma, São Luís, Brazil; ^8^Departmento de Biologia, Instituto Federal do Maranhão, São Luís, Brazil

**Keywords:** *Candida* infections, *Candida*, antifungals, resistance, alternative antifungal drugs

## Abstract

The *Candida* genus comprises opportunistic fungi that can become pathogenic when the immune system of the host fails. *Candida albicans* is the most important and prevalent species. Polyenes, fluoropyrimidines, echinocandins, and azoles are used as commercial antifungal agents to treat candidiasis. However, the presence of intrinsic and developed resistance against azole antifungals has been extensively documented among several *Candida* species. The advent of original and re-emergence of classical fungal diseases have occurred as a consequence of the development of the antifungal resistance phenomenon. In this way, the development of new satisfactory therapy for fungal diseases persists as a major challenge of present-day medicine. The design of original drugs from traditional medicines provides new promises in the modern clinic. The urgent need includes the development of alternative drugs that are more efficient and tolerant than those traditional already in use. The identification of new substances with potential antifungal effect at low concentrations or in combination is also a possibility. The present review briefly examines the infections caused by *Candida* species and focuses on the mechanisms of action associated with the traditional agents used to treat those infections, as well as the current understanding of the molecular basis of resistance development in these fungal species. In addition, this review describes some of the promising alternative molecules and/or substances that could be used as anticandidal agents, their mechanisms of action, and their use in combination with traditional drugs.

## Introduction

*Candida* species, opportunistic pathogens, are a major cause of morbidity and mortality worldwide and thus represents a serious threat to public health (Pfaller et al., 2014; Matthaiou et al., 2015; Pappas et al., 2016). Further, *Candida* species can cause vaginitis, oral candidiasis, cutaneous candidiasis, candidemia, and systemic infections (Wächtler et al., 2012). Candidemia is the most frequent hospital infection accounting for up to 15% of bloodstream infections, and *Candida* species are the main causative agents in 50–70% of systemic fungal infections (Cornely et al., 2012; Lionakis and Netea, 2013; Barchiesi et al., 2016).

*Candida albicans* is the pathogenic species most frequently isolated. However, other species such as *C. glabrata, C. tropicalis*, *C. parapsilosis*, *C. krusei*, *C. famata, C. guilliermondii*, and *C. lusitaniae* have been increasingly isolated, mainly in human immunodeficiency virus (HIV)-infected individuals (Brunke and Hube, 2013; Ferreira et al., 2013; Mayer et al., 2013; Patil et al., 2015; Barchiesi et al., 2016).

The pathogenesis of *Candida* species is poorly understood, and the rate of infections is increasing rapidly. Further, a steady increment in resistance to traditional antifungal has resulted in the need to control *Candida* infections through early diagnosis and prevention of candidiasis.

Among the available antifungal agents, azoles are the preferred and most frequently used drugs for treatment of *Candida* infections. Depending on the type of infection, the anatomical site in which it occurs and the sensitivity profile of species, other antifungals can also be used. Among these, there are polyenes, echinocandins, nucleoside analogs and allylamines (Pfaller et al., 2010a, 2013; Pappas et al., 2016). Fluconazole (FLZ), a type of azole, is often preferred in treatments of *Candida* infections because of its low cost and toxicity, in addition to availability in varied formulations (Pfaller et al., 2010b). However, there are many reports in the literature on the development of resistance among *Candida* species, especially in relation to azoles, which is essential for the determination of resistance mechanisms presented by fungi with the objective of developing new classes of antifungal for treatment of *Candida* infections.

The need of the hour includes the development of a more effective therapy, since the phenomenon of resistance caused the appearance of new fungal infections, in addition to facilitating the resurgence of the existing ones. In this way, the control of *Candida* infections is a challenge in the modern clinic. The design of new drugs from the traditional ones used in the clinic and the identification of new molecules with antifungal potential for the manufacture of new drugs, more effective and less toxic, are fundamental to face the challenge.

The present review examines infections caused by *Candida* species and describes our current understanding of the molecular basis of resistance development in these fungal species. In addition, this review describes some of the promising alternative molecules and/or substances that are effective pharmaceuticals for treating fungal infections and could be used as anticandidal agents, as well as their mechanisms of action.

## Candida Infections

Fungal infections are considered a serious health problem, especially in people with some impairment in the immune system and are a main cause of morbidity and mortality worldwide (Vallabhaneni et al., 2016). In the last two decades, fungal infections have shown a significant increment. This high incidence has been related to factors such as the increase in the number of patients with compromised immune system, (Ortega et al., 2010; Junqueira et al., 2012; Li et al., 2013; Terças A.L.G. et al., 2017), the increasing number of patients receiving hyperalimentation through catheters or probes and use of broad-spectrum antibiotics (Bouza and Munoz, 2008). The rising number of patients requiring organ transplantations, as well as those with leukemia and diabetes also contributes to this phenomenon (Razzaghi-Abyaneh et al., 2014).

The most frequent fungal disease affecting populations in the world is candidiasis (Lewis et al., 2012; Ferreira et al., 2013; Kwamin et al., 2013; Mayer et al., 2013; Tsai et al., 2013; Vázquez-González et al., 2013). There are several types of candidiasis as mucosal candidiasis, cutaneous candidiasis, onychomycosis and systemic candidiasis (Calderone and Fonzi, 2001; Kim and Sudbery, 2011; Wächtler et al., 2012). An important fact is that candidiasis is an infection that can affect both immunocompromised and healthy people (Li et al., 2006; Raman et al., 2013). Candidemia is another infection due *Candida* spp. and is the most relevant and prevalent nosocomial fungal infection associated with a high mortality rate (up to 49%) in patients with a compromised immune system (Pfaller and Diekema, 2007; Sardi et al., 2013). The association of *Candida* with bloodstream infections depends on patient’s condition, age, and geographic region. Candidemia is such an important infection that in 10–40% of cases it is associated with sepsis or septic shock while *Candida* species as main agent of sepsis or septic shock are responsible for no more than 5% of the total number of cases (Guery et al., 2009).

Many species recovered from human samples have been identified as belonging to the genus *Candida* that almost half has implicated in serious infections. *C. albicans* continues to be the most prevalent species, representing the majority of isolates of fungal infections (Delgado et al., 2009; Hise et al., 2009; Junqueira et al., 2012; Li et al., 2013; Terças A.L.G. et al., 2017). However, the prevalence of other *Candida* species has increase substantially. These species are *C. parapsilosis, C. tropicalis*, *C. krusei*, *C. glabrata*, *C. guilliermondii*, *C. orthopsilosis, C. metapsilosis, C. famata*, and *C. lusitaniae* (Sant’Ana Pde et al., 2002; Li et al., 2013; Kaur et al., 2016).

*Candida albicans* is a species that presents high degree of flexibility, being able to grow in extremely different environments regarding to the availability of nutrients, temperature variation, pH, osmolarity, and amount of available oxygen (Paramythiotou et al., 2014). This fact associated with the high resistance capacity of the species to antifungals, their virulent features, capability of forming biofilms with other species (Álvares et al., 2007; Shoham and Marr, 2012) make the genus *Candida* a serious risk to human health (Soll, 2008). Thus, *Candida* species are highly adaptable and possess numerous strategies to survive favors that might affect their overgrowth and change their susceptibility profiles.

In addition, it is difficult to identify specific *Candida* species, which may delay the use of precise therapeutics. For instance, microbiological tests using specific culture media do not differentiate many species of *Candida*. Often, it takes several days to obtain antifungal susceptibility information for *Candida* species (Clancy and Nguyen, 2013). Although there are newer molecular techniques available for rapid yeast detection, such as fluorescence *in situ* hybridization (PNA-FISH), commercially available equipment for analysis does not differentiate between *C. albicans* and *C. parapsilosis*, *C. glabrata*, or *C. krusei*. These facts are relevant because increased mortality rate is associated with delays in initiating adequate antifungal therapy (Garey et al., 2006; Bassetti et al., 2014).

## Traditional Agents and Mechanisms of Action

**FIGURE 1:**
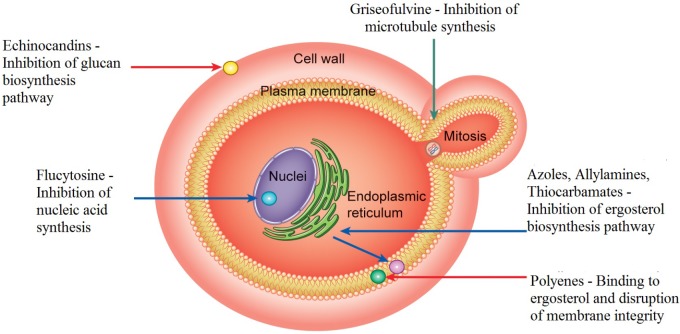
Mechanisms of action of traditional antifungal agents on cellular targets. Azoles inhibit the ergosterol synthesis in the endoplasmic reticulum of the fungal cell. They act by interfering with the enzyme lanosterol 14-α-demethylase, involved in the transformation of lanosterol into ergosterol. Polyenes act in the fungal membrane by binding to ergosterol and causing disruption of the membrane structure promoting extravasation of intracellular constituents and, consequently, cell death. Flucytosine inhibits the thymidylate-synthetase enzyme interfering with DNA. Echinocandins inhibit (1,3) β-D-glucan synthase, thereby preventing glucan synthesis, which is present in the cell membrane of fungi. Allylamines and thiocarbamates inhibit the enzyme squalene-epoxidase, which participates in the synthesis of ergosterol. Griseofulvin acts by disrupting spindle and cytoplasmic microtubule production, thereby, inhibiting fungal mitosis.

Fungal cells, like human cells, are eukaryotic; both cell types are targeted by antifungal compounds, resulting in considerable side effects in patients and fewer available targets for drug action. Since the 1990s, there has been an increasing, but limited, discovery of antifungal agents (Sardi et al., 2013; Paramythiotou et al., 2014). These drugs include azoles, that inhibit ergosterol biosynthesis [FLZ, itraconazole (ITC), ketoconazole (KTC), miconazole and clotrimazole]; polyenes [amphotericin B (AMB) and nystatin]; allylamines; thiocarbamates; morpholines; 5-fluorocytosine, a deoxyribonucleic acid (DNA) analog; and echinocandins (for instance, caspofungins) (Pappas et al., 2009; Spampinato and Leonardi, 2013). Three cellular components of fungi are targeted by these drugs. In endoplasmic reticulum of the fungal cell, azoles inhibit ergosterol biosynthesis by interfering with the enzyme lanosterol 14-α-demethylase, involved in the transformation of lanosterol into ergosterol, component that is part of the plasma membrane structure of the fungus (**Figure 1**). Thus, accumulation of 14-α-methyl-3, 6-diol, a toxic compound, will occur. As the concentration of ergosterol is reduced, the cell membrane structure is altered, thereby inhibiting fungal growth (Sanguinetti et al., 2015).

Azoles comprise a 5-member azole ring containing two (imidazole) or three nitrogen atoms (triazole) attached to a complex side chain (Georgopapadakou, 1998; Groll et al., 2003). The azole family of compounds includes the imidazoles (KTC, miconazole, econazole and clotrimazole) and triazoles (FLZ, ITC, and voriconazole, which is a synthetic triazole derivative of FLZ of second-generation), and posaconazole (hydroxylated analog of itraconazole) (Kontoyiannis et al., 2003; Maubon et al., 2014). Ergosterol is similar to cholesterol present in plasma membrane of animal cells; however, most antimycotic agents that target ergosterol binding or synthesis do not cross-react with host cells because of sufficient structural differences between these molecules (Spampinato and Leonardi, 2013).

Amphotericin B and others polyenes act in fungal membrane by binding to ergosterol and causing disruption of the membrane structure, which promotes extravasation of intracellular constituents such as potassium, magnesium, and sugars and, consequently, cell death (Perman et al., 2009; Mesa-Arango et al., 2012; **Figure 1**).

Flucytosine (5-FC) is a pyrimidine analog with fungistatic properties that enters the fungal cell through cytosine permease and inhibits the thymidylate-synthetase enzyme interfering with DNA. 5-FC can also be converted to 5-fluorouracil which in turn can be phosphorylated to 5-fluorodeoxyuridine monophosphate. This one being also phosphorylated can be incorporated into RNA molecules, thus interfering with the cell translation process (Cuenca-Estrella, 2010; Spampinato and Leonardi, 2013; Maubon et al., 2014; **Figure 1**). Further, 5-fluorodeoxyuridine is associated with considerable toxicity (Patil et al., 2015). Clinical use of 5-FC is preferred in association with AMB (Sanglard et al., 2009; Nett and Andes, 2016; Prasad et al., 2016), since the use of 5-FC alone induces stronger side-effects, such as hepatic impairment, interference with bone marrow function, and rapid occurrence of resistance especially among *Candida* species (Groll et al., 1998; Nett and Andes, 2016; Prasad et al., 2016).

According to the type of infection and the sensitivity/resistance profile of the isolates, as well as the site of origin of the samples all antifungal agents may be used with varying efficiency (Pfaller et al., 2010a). AMB is considered the gold standard drug for most mycoses that affect patients at risk (Mesa-Arango et al., 2012). However, AMB has high toxicity, which limits its use. Nephrotoxicity is a main effect resulting from AMB administration (Mesa-Arango et al., 2012; Nett and Andes, 2016). To minimize this problem and to increase the effectiveness of treatment, some formulations have been developed. Liposomal AMB (Ambisome^®^) allows for lower absorption of AMB by the reticuloendothelial system, which results in greater permanence in the bloodstream. A lipid complex of AMB (Abelcet^®^) comprises 50% AMB and 50% lipid compound and AMB in a colloidal dispersion (Amphocil^®^/Amphotech^®^, formed by a stable complex with cholesterol sulfate). However, the high cost of these formulations has limited their use (Kontoyiannis et al., 2003; Mesa-Arango et al., 2012; Paramythiotou et al., 2014; Nett and Andes, 2016).

Azoles represent the class of antifungals with the highest number of drugs. Azoles have fungistatic properties that affect cell growth and proliferation; a large amount of accumulated toxic sterols can eventually lead to fungal cell death (Shapiro et al., 2011; Prasad et al., 2016). Such agents are preferred in the treatment of candidemia (Spampinato and Leonardi, 2013; Maubon et al., 2014) and candidiasis. Among azoles, miconazole and KTC (imidazoles) first emerged and were the only drugs available for systemic use, with KTC being the first alternative to AMB (Groll et al., 1998; Seyedmousavi et al., 2017). Then triazoles as itraconazole and FLZ have emerged, more effective and better tolerated than KTC (Dismukes, 2000).

Fluconazole is the drug of choice for most *Candida* infections (Pfaller et al., 2010b; Patil et al., 2015) and is the most recommended antifungal agent, attributable to its low cost, for use in invasive candidiasis in patients who have not previously been medicated with azole antifungal agents (Shoham and Marr, 2012; Paramythiotou et al., 2014).

Azole antifungals have limitations to their use, although they are generally well-tolerated. Limitations include adverse effects such as hepatotoxicity and the emergence of resistance among fungal isolates (Carrillo-Muñoz et al., 2006). Azoles can be toxic because they act as substrates or inhibitors of several enzymes such as cytochrome P450 enzymes. Further, these limitations provide motivation for improving this class of antifungal agents (Nett and Andes, 2016).

Alterations in triazole molecule gave rise to voriconazole (structurally related to FLZ) and posaconazole (related to ITC), a second generation of antifungals. Both are available for systemic therapy and have been shown to have better specificity and antifungal potency than that of first generation triazoles (Nett and Andes, 2016).

A new class of drugs, the echinocandins, has been shown to have fungicidal effects in all *Candida* species (Nett and Andes, 2016). The echinocandins include caspofungin, micafungin, and anidulafungin (Grossman et al., 2014; Koehler et al., 2014; Paramythiotou et al., 2014). Echinocandins inhibit (1,3) β-D-glucan synthase, thereby preventing glucan synthesis, which is present in the cell membrane of fungi (**Figure 1**). β-D-Glucan synthase inhibition depletes glucan polymers in fungal cells, resulting in an abnormal cell wall that is weak and unable to resist osmotic stress (Chen and Sorrel, 2007; Kuse et al., 2007). This class of drugs has certain advantages attributable to its effects on the fungal cell wall, including a lower risk of side effects since animal cells do not have this structure. Further, echinocandins can be used in cases of azole-antifungal resistance (Spampinato and Leonardi, 2013; Grossman et al., 2014; Maubon et al., 2014; Paramythiotou et al., 2014).

Allylamines (terbinafine and naftifine) and thiocarbamates inhibit the enzyme squalene-epoxidase, which participates in the synthesis of ergosterol and is encoded by the *ERG1* gene (**Figure 1**). Inhibition of squalene-epoxidase leads to membrane rupture and accumulation of squalene. Allylamines effects can also prevent the production of other sterol derivatives.

Another antifungal is the tricyclic spirodiketone griseofulvin that acts by interfering the cytoplasmic microtubule production, disrupting spindle formation and, thereby inhibiting fungal mitosis (**Figure 1**). Griseofulvin was isolated from *Penicillium griseofulvum*, (Francois et al., 2005).

Certain pharmacological strategies have been developed to minimize toxicity and resistance. Development and use of new antifungal formulas (liposomal AMB, AMB lipid complexes, AMB colloidal dispersions, and AMB lipid nanosphere formulations), itraconazole, and β-cyclodextrin itraconazole is one strategy. Others include combination therapies of antifungal compounds (for example, AMB + 5-FC, FLZ + 5-FC, AMB + FLZ, caspofungin + liposomal AMB, and caspofungin + FLZ) (**Table 1**) and nanostructuring of conventional antifungal agents (Amaral and Felipe, 2013; Spampinato and Leonardi, 2013; Stiufiuc et al., 2015; Souza and Amaral, 2017).

**Table 1:** Various regimes of combinatorial antifungal therapy showing better efficacy in combination than that of independent drugs.

Combination of antifungals	Target	Reference
AMP B + Posaconazole AMP B + Caspofungin	*Candida* biofilms	Bink et al., 2011; Rodrigues et al., 2014.
Micafungin + Fluconazole Micafungin + Voriconazole Micafungin + AMP B	*Candida* infections	Serena et al., 2005; Espinel-Ingroff, 2009.
Flucytosine + Voriconazole	*Candida* infections	Bink et al., 2011
Minocycline + Fluconazole	*Candida albicans* biofilms	Bink et al., 2011
Posaconazole + Caspofungin	*Candida* infections	Chaturvedi et al., 2011; Chen et al., 2013
Terbinafine + Azole	*Candida* growth	Barchiesi et al., 1997; Perea et al., 2002a
Echinocandin + Azole	Invasive candidiasis	Cui et al., 2015
AMP B + Flucytosine	Invasive candidiasis	Pappas et al., 2016

*AMP B, amphotericin B.*

Chaturvedi et al. (2011) evaluated the sensitivity profile of reference and clinical samples of *C. albicans*, *C. glabrata*, and *C. parapsilosis* in relation to antifungals like azoles and equinocandinas. They found that despites clinical isolates had relatively high azole and echinocandin MICs, some synergistic combinations were found for AMB- posaconazole against *C. glabrata* and AMB- anidulafungin and AMB- caspofungin against *C. parapsilosis* by both visual and spectrophotometric readings. Chen et al. (2013) a potential therapeutic applicability for posaconazole and caspofungin combinations in the future. Their studies reported that posaconazole exhibits *in vitro* and *in vivo* synergy with caspofungin against drug susceptible or resistant *C. albicans* strains (derived echinocandin-resistant mutants).

Pappas et al. (2016) drew attention to the fact that a combination of liposomal AmB, 5 mg/kg daily, and flucytosine, 25 mg/kg 4 times daily, may be considered as salvage therapy in patients who have not had a clinical response to initial AmB therapy in cases of central nervous system infections by fungus in neonates.

Examples of some others publications on combination therapy between different antifungal drugs are shown in **Table 1**.

However, among traditional antimycotic drugs, none has all the qualities required for an ideal agent (Wong et al., 2014). All drugs have at least one of the following restrictions: they do not have a broad spectrum of action, some are fungistatic, and others have high toxicity and low bioavailability with significant side effects in patients undergoing therapeutic regimens (Petrikkos and Skiada, 2007; Safdar et al., 2010; Lewis et al., 2012; Vollenbroich et al., 2014; Bayhan et al., 2015). Therefore, limitations of treatment and drug resistance (Canuto and Rodero, 2002; Petrikkos and Skiada, 2007; Mukherjee et al., 2011; Tscherner et al., 2011) associated with pathogenicity of the clinical isolates support the urgent need to identify substances that are more effective, with new mechanisms of action in the fight against *Candida* infections.

## Mechanisms of Cellular and Molecular Antifungal Resistance

There are three types of antifungal resistance, including a primary or intrinsic form that exists prior to antifungal exposure. The second type is an acquired form that occurs after antifungal exposure and may be reversible, attributable to transient or non-reversible adaptation resulting from several genetic alterations. A clinical form refers to unfavorable outcomes in patients despite antifungal therapy and is directly linked to primary or secondary resistance (Cowen et al., 2015).

**FIGURE 2:**
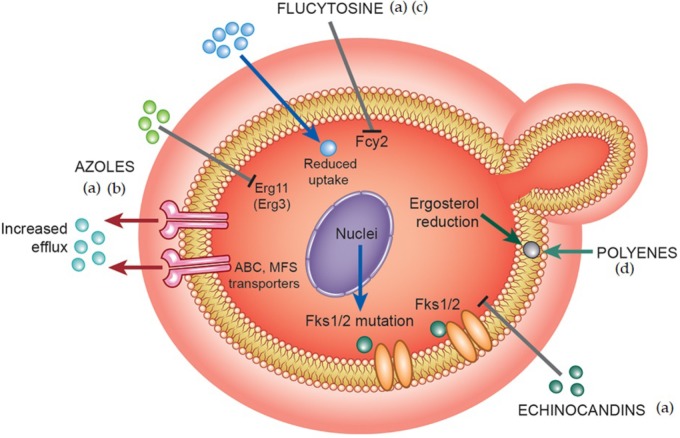
Different mechanisms of multidrug resistance adopted by fungal cells. The main mechanisms of drug resistance against azoles, polyenes, echinocandins, and flucytosine include: (a) alteration of the enzyme target (genes encoding ergosterol biosynthetic pathway enzymes – AZOLES, glucan synthases – ECHINOCANDINS, cytosine deaminase or uracil phosphoribosyltransferase – FLUCYTOSINE) leading to poor binding of toxic drugs to its enzyme target sites, (b) overexpression of drug efflux proteins leading to increased efflux – AZOLES, (c) changes in membrane property/composition affecting normal drug import – FLUCYTOSINE, (d) Reduction of sterols in plasma membrane – POLYENES. Colored balls mean antifungal molecules.

Populations are increasingly at risk of fungal infections, resulting in an increased use of antifungal agents. Consequently, higher minimum inhibitory concentrations (MIC) for antifungals against *C. albicans* strains have been observed and may be related to therapeutic failures. In addition, some non-*albicans Candida* (NAC) species have inherent resistance to azoles (Oxman et al., 2010; Lortholary et al., 2011; Fothergill et al., 2014). Low-dose prophylactic administration of azole derivatives, such as FLZ, for prolonged periods to prevent the occurrence of opportunistic infections in immunosuppressed patients also results in resistant phenotypes (Siikala et al., 2010; Rautemaa and Ramage, 2011). These facts are likely to collaborate to the increased incidence of fungal infections. Resistance to polyenes (AMB) in *C. albicans* is less common and is associated with the substitution of ergosterol with a precursor molecule or a general reduction of sterols in the plasma membrane (Kanafani and Perfect, 2008) (**Figure 2**). Enzymes such as Δ_5,6_-desaturase, encoded by *ERG3* gene, and C-8 sterol isomerase, encoded by *ERG2* gene participate in ergosterol biosynthesis and have the main alterations related to AMB resistance. These enzymes influence the amount of ergosterol required for the action of polyenes because the mutations are responsible for modifications in sterol content (Sheikh et al., 2013). For instance, Δ_5,6_-desaturase when mutated converts fecosterol to episterol which has low affinity for AMB. Another likely AMB-resistant mechanism is the reduction of oxidative damage via enhanced catalase activity (Kanafani and Perfect, 2008).

Possible mechanisms for cellular and molecular resistance to FLZ in *C. albicans* are described. The first is related to induction of multi-drug pumps, which decrease the concentration of drug available for the target enzyme, 14-α-demethylase, in fungal cells (Kanafani and Perfect, 2008) (**Figure 2**). There are two types of active transporters in *C. albicans*, including those encoded by the *Candida* drug resistance*-CDR* genes (Cdr1 and Cdr2) and those encoded by the multidrug resistance*-MDR1* genes. Cdr1- and Cdr2-type pumps are ATP-binding cassette (ABC) transporters, and Mdr1 is a major facilitator superfamily (MFS)-type pump that transports solutes from different sides of the fungal cell plasma membrane. Overexpression of transporters encoded by *CDR* genes confers cross-resistance to various azole-derived compounds, while overexpression of those transporters encoded by *MDR1* genes is responsible for FLZ resistance. Superexpression of these transporters prevents accumulation of the drug in the intracellular compartment (Kanafani and Perfect, 2008; Pfaller, 2012) (**Figure 2**).

A second mechanism of resistance involves modification of the target enzyme encoded by the *ERG11* gene, also known as cytochrome P_450_ lanosterol 14 α-demethylase (Cyp51) (**Figure 2**). Mutations in this gene prevent azoles from binding to enzyme sites (Marichal et al., 1999; Flowers et al., 2015). Another mechanism of resistance to azoles is related to substitution of ergosterol by another sterol. Mutations in the *ERG3* gene does not convert 14-α-methylfecosterol into 14-α-methyl-3,6-diol. This substitution causes azoles to have no fungistatic effects on the fungal cell membrane (Sanguinetti et al., 2015).

Two possible mechanisms of resistance to echinocandins have been reported. The first deals with point mutations in gene that encodes the major subunit of the glucan synthase enzyme (Fks subunit) (**Figure 2**). These mutations occur only in two regions of the gene (known as “hot-spot”), are dominants and can provide resistance to all echinocandin (Perlin, 2015). The consequence is that these mutations lead to the production of high MIC values. For instance, *C. parapsilosis* and *C. guilliermondii* present MIC values 4- to 100-fold greater compared to those observed for *C. albicans.* The *FKS1* mechanism extends to other NAC species such as *C. tropicalis*, *C. parapsilosis*, *C. glabrata*, *C. krusei*, *C. guilliermondii*, and *C. dubliniensis* that show the same mutations as those of *C. albicans* (Katiyar et al., 2006; Perlin, 2011). In that way, *FKS1*-mediated resistance mechanisms can be pervasive in the fungal kingdom because it is suggested that it can be responsible for a reduced inherit sensitivity of certain *Candida* species and molds (Perlin, 2007).

The second mechanism of resistance involves the response to adaptation stress. When there is an inhibition of production of fungal cell wall component, microorganism is capable of increasing the production of another one. Some research showed that many *Candida* species respond to the inhibition of Fks synthesis producing high amounts of chitin (Chamilos et al., 2007; Shields et al., 2011). Chamilos et al. (2007), studying *Candida* bloodstream isolates from cancer patients, observed a process known as the paradoxical effect in some isolates of *Candida*, that is, isolates are capable of growing in the presence of high concentrations of echinocandins (above the MIC). This phenomenon was strikingly absent in *C. glabrata* isolates, but was well evidenced in *C. parapsilosis*, *C. tropicalis*, and *C. krusei* ones (Chamilos et al., 2007).

Resistance to 5-FC can be of two types: primary, occurring via cytosine permease (encoded by the *FCY2* gene) with decreased drug uptake (Sanglard and Odds, 2002) (**Figure 2**); secondary, related to alterations in cytosine deaminase (encoded by *FCY1*) or uracil phosphoribosyltransferase (encoded by *FUR1*) enzymes activities. Cytosine permease is responsible by conversion of 5-FC to 5-fluorouridine or to 5-fluorouridine monophosphate (5-FUMP) (Kontoyiannis and Lewis, 2002; Espinel-Ingroff, 2008) Resistance is easily developed in fungal isolates from patients who are receiving the drug. However, most of these mechanisms have only been observed in others species of *Candida*, but not in *C. albicans* (Papon et al., 2007). Therefore, other molecular mechanisms related to resistance to 5-CF must exist, playing relevant role in fungal resistance (Schwarzmuller et al., 2014).

Costa et al. (2015) recently reported a relation between arginine metabolic enzymes and 5-FC resistance, suggesting that 5-FC resistance requires somehow high arginine production. Accordingly, L-arginine concentrations in some body fluids in healthy individuals, as vaginal fluid (Gregoire et al., 1959) or human plasma (Armengou et al., 2003) can be as high as 0.1 mM. The molecular mechanisms involved in arginine and 5-FC resistance relationship have not been clarified, but the results obtained by the authors highlight the significance of a new possibility of fighting resistance to 5-FC.

### Mutations in Genes Associated With Resistance to Azoles in *Candida* Species

Studies aimed at elucidating the molecular mechanisms responsible for developing resistance to *Candida* species have predominantly focused on resistance to azoles, as they are the most commonly used drugs clinically (Cernicka and Subik, 2006; Gualco et al., 2007). Thus, the main research targets include the *CDR1*, *CDR2*, and *MDR1* genes (Puri et al., 1999; Yang and Lo, 2001; Morschhäuser et al., 2007; Tsao et al., 2009). In addition, mutations in transcription factors associated with the *CDR1* and *CDR2* genes (Chen et al., 2004; Coste et al., 2006; Wang et al., 2006), specific mutations or superexpression of *ERG11* genes, and mutations in the *ERG5* or *ERG3* genes (also involved in ergosterol biosynthesis), have been identified. Most of them were related to FLZ resistance (Sanglard et al., 2003; Lo et al., 2005; Martel et al., 2010).

### Mutations Associated With Efflux Pump Genes (*CDR1*, *CDR2*, and *MDR1*) and Transcription Factors

An important and potent mechanism of multi-drug resistance (MDR) in fungi is the intracellular accumulation of antifungals by increased efflux of drugs (Prasad and Kapoor, 2005; Prasad and Rawal, 2014). In *C. albicans*, overexpression of genes encoding transporters proteins, mainly *CDR1* and *CDR2* genes (encoding Cdr1 and Cdr2, respectively, which are ABC multidrug transporter proteins) or *MDR1* gene (encoding the MFS efflux pump protein Mdr1) is considered the main mechanism responsible for antimycotic resistance in *Candida* isolates (Franz et al., 1998; Lopez-Ribot et al., 1998; Lyons and White, 2000; Wirsching et al., 2000; Kusch et al., 2004; Niimi et al., 2004; Prasad and Kapoor, 2005; Prasad and Rawal, 2014). Increased production of the Cdr1 transporter is responsible for FLZ, KTC, and ITC resistance. In contrast, expression of the *Candida* drug resistance protein 2 (Cdr2p) is related to FLZ and ketoconazole resistance, but does not affect resistance to itraconazole (Tsao et al., 2009). There are related multidrug transporters with MDR function in NAC species such as those in *C. glabrata* (CgCdr1, CgCdr2 and Snq2) (Miyazaki et al., 1998; Sanglard et al., 1999; Torelli et al., 2008) and in *C. krusei* (*ABC1)* (Katiyar and Edlind, 2001).

Azole resistance in *C. glabrata* probably is also related to upregulation of homologous transporter genes *CgCDR1* and *CgCDR2* (Sanglard et al., 1999, 2001; Bennett et al., 2004), and genetic evidence has been provided that supports a role for multidrug transporters in azole resistance in *C. glabrata* (Sanglard et al., 1999).

Looi et al. (2005) investigated expression of the *C. albicans* and *C. glabrata CDR1* and *MDR1* genes associated with azole resistance in patients with vaginitis. There was overexpression of genes to varying extents in all *Candida* isolates tested and this result was correlated with the degree of resistance, as evidenced by antifungals MICs. The authors also observed that in one *C. albicans* resistant isolate there was overexpression of messenger RNA for Mdr1 after superexpression of Cdr1, which suggests a synergism between these drug efflux pumps proteins. DNA sequence analysis of the *CDR1* promoter region also suggests there are several point mutations in resistant clinical isolates that are not present in susceptible isolates. Thus, this region is important for binding of transcription factors and for increasing the affinity of activators responsible by *CDR1* expression in drug resistant isolates.

Sanguinetti et al. (2005) evaluated the molecular mechanisms of resistance in 29 nosocomial isolates of *C. glabrata* recovered during 3 years of study; of these, most were resistant to FLZ. Quantitative real-time PCR analyses provided evidence that azole resistance in these isolates probably was due the upregulation of genes *CgCDR1*, *CgCDR2*, and *CgSNQ2*, encoding efflux proteins in *C. glabrata*.

Katiyar and Edlind (2001) identified two homologous ABC transporter genes (*ABC1* and *ABC2*) in *C. krusei* previously described for *C. albicans*. When cultures of *C. krusei* were exposed to imidazole and cycloheximide, *ABC1* gene was upregulated. Lamping et al. (2009) showed that *ABC1* is involved in the inherent resistance of *C. krusei* to azoles. In *C. parapsilosis* authors have shown that *MRR1* is involved in resistance to FLZ (Souza et al., 2015; Zhang et al., 2015); however, in *C. tropicalis*, efflux pumps genes related to azoles resistance have not yet been identified (Barchiesi et al., 2000).

Gołąbek et al. (2015) also studied the expression of *CDR1*, *CDR2*, and *MDR1* genes in 120 strains of *C. albicans* (60 resistant and 60 azole susceptible) obtained from clinical samples and observed that the expression of Cdr1, Cdr2, and Mdr1 was higher in azole-resistant strains than that in sensitive strains. Several transcription factors have been identified as responsible for upregulating *CDR1*, *CDR2*, and *MDR1* genes, with several serving as positive *MDR1* regulators (Coste et al., 2004; Wang et al., 2006) and others as negative *MDR1* regulators (Chen et al., 2009).

Overexpression of the *CDR1* and *CDR2* genes has been suggested to influence the relationship between susceptibility and resistance to azoles and AMB. According to Ren et al. (2014), azole-resistant strains of *C. albicans* that overexpress *CDR1* and *CDR2* are hypersensitive to AMB. In contrast, knockout strains for the *CDR1* and *CDR2* genes are resistant to AMB, suggesting that the ergosterol content determines sensitivity to both azoles and AMB in *C. albicans*, and that there is an inverse susceptibility to these drugs that is directly associated with Cdr1 and Cdr2 transporters. The authors also suggest a new therapeutic approach for administering alanine phosphoricin B in situations of fungal resistance to azoles rather than increasing the administered dose of the azole agent.

Major facilitator superfamily (MFS) transporters were first identified in *C. albicans* and are the second major superfamily of transporters also related to drug efflux in this species (Saier et al., 1999; Gaur et al., 2008). MFS proteins consist of one polypeptide chain two three-dimensional regions with independent functions (“domains”) having six transmembrane alpha helical spanners (TMSs). There are two types of MFS proteins, including DHA1 (drug:HC antiporter-1) with 12 TMSs, and DHA2, which has 14 TMSs. The main multidrug protein of *C. albicans* is MDR1 from the DHA1 subfamily. Homologs of *CaMDR1* were identified in *C. glabrata* (*CgMDR1*) and in *C. dubliniensis* (*CdMDR1*). *CgMDR1* is constitutively expressed and confers specific resistance to FLZ in *C. glabrata*; therefore, this phenomenon could explain the intrinsic resistance of this yeast to triazoles (Moran et al., 1998; Sanglard et al., 1999). In *C. dubliniensis* overexpression of *CdMDR1* is considered a main mechanism of FLZ resistance in isolates of this species (Moran et al., 1998).

### Mutations Associated With the *ERG11* Gene

In *C. albicans*, a 1587 bp gene encodes Erg11 protein. Erg11 has 595 amino acids. Up to now, approximately more than a hundred non-synonymous point mutations have been identified in clinically resistant isolates (Noel, 2012; Strzelczyk et al., 2013). Interestingly, most of these substitutions occur in 3 regions ranging from 105 to 165, 266 to 287, and 405 to 488 amino acids (“hot spots”) instead of being randomly dispersed (Marichal et al., 1999; Wang et al., 2009). However, many of the identified mutations are not resistant and are considered genetic polymorphisms occurring in living organisms. Few of these mutations have been demonstrated to support azole resistance (Noel, 2012; Strzelczyk et al., 2013). *ERG11* genetic polymorphisms should be considered in the rational design of novel azole-derived drugs, attributable to certain polymorphisms identified in the gene that do not necessarily characterize changes in the amino acids and three-dimensional structure of proteins, and therefore, do not reduce the affinity between azolic components and the protein. Thus, mapping all *ERG11* amino acid changes involved in azole resistance could help in the design of new azole antifungals with potent activity against resistant strains.

Recent studies corroborate these facts. For instance, Morio et al. (2010) investigated the susceptibility of FLZ, ITC and voriconazole in isolates of *C. albicans* and verified 23 distinct substitutions, 2 of which were suspected as being involved in azole resistance. Gołąbek et al. (2015) verified 19 changes in the *ERG11* gene sequence and found that five alterations occurred in azole resistant strains only (A530C, G622A, G1309A, A1167G, and A1230G). Further, 33% of azole-resistant strains were characterized by the simultaneous presence of the A530C, G622A, and A1167G mutations. Caban et al. (2016) identified 21 specific mutations in the *ERG11* gene, two of which were significantly associated with drug resistance, including a nucleotide substitution at position 798, which was related to an increase in drug resistance, and a silence mutation at position 1440, which significantly decreased the chance of a strain being resistant to drugs.

*ERG11* mutations conferring azole resistance in *C. tropicalis* (Vandeputte et al., 2005; Jiang et al., 2013), *C. krusei* (Ricardo et al., 2014), *C. dubliniensis* (Perea et al., 2002b), and *C. parapsilosis* (Grossman et al., 2015) clinical isolates have also been described; however, there is no relate about these mutations in *C. glabrata* (Gonçalves et al., 2016).

In addition to mutations that directly affect the *ERG11* gene, there are changes in transcription factors associated with this gene that may also affect its expression and, consequently, the biosynthesis pathway of ergosterol. The *C. albicans* Upc2p transcription factor (*CAUPC2*) gene is among the regulators of *ERG11* gene expression.

### Overexpression of *ERG11*

It is known from the literature that overexpression of *ERG11* gene, or maybe its upregulation, are responsible for the resistance to azole agents. Accordingly, different methods have been used to measure the level of *ERG11* expression by detecting and quantifying its mRNA. A 3- to 20-fold increase in mRNA production was observed in resistant strains (Sanguinetti et al., 2015).

Two independent mechanisms have been shown to drive *ERG11* overexpression. One is related to a chromosomal mutation (duplication) of *ERG11* gene. This phenomenon was demonstrated first in *C. glabrata* isolates (Marichal et al., 1997); this occurs when an isochromosome is formed in the region having the *ERG11* gene. In that way, this specific region will now have two copies of the left arm of chromosome 5, duplicating the chromosome (Selmecki et al., 2006). The second depends on a transcription factor that regulates the ergosterol biosynthesis (Upc2p, coded by the *CaUPC2* gene) identified in *C. albicans*. Upc2p recognizes and is specifically bound to the promoters (the well-known SRE box, of sterol response element) of different *ERG* genes that activate gene transcription (Noel, 2012).

Antifungal drugs, including FLZ, induce the expression of *CaUPC2* gene, and the Upc2p transcription factor upregulates *ERG2* and *ERG11* gene expression when *C. albicans* is grown under azole drug pressure (Allen et al., 2015). Studies have shown that strains that have undergone homozygous deletion of the *CaUPC2* gene are hypersensitive to several drugs and accumulate significantly less cholesterol, suggesting a decrease in ergosterol in these strains (Silver et al., 2004).

Different strains of *C. albicans* and probably other *Candida* species express different *UPC2* alleles encoding for transcription factors of different strength. In addition, three gain of function mutations (A643T, A643V, and G648D) have been characterized in sequential clinical isolates overexpressing *ERG11*. Upc2 proteins act constitutively and lead to loss of sensitivity to azoles (Noel, 2012).

## Alternative Agents as Anticandidal Agents

Increased drug resistance in fungi is a problem that cannot be avoided, particularly for FLZ, which is the preferred antifungal for treating candidiasis in acquired immunodeficiency syndrome (AIDS) patients (Siikala et al., 2010; Rautemaa and Ramage, 2011). Moreover, there are fungi that have intrinsic resistant to antifungal agents commonly used in the clinic (Sanglard, 2016). In addition, biofilms, an ordinary virulence property of fungi, has as main characteristic the capacity of resistance to drugs (Chandra et al., 2005; Seneviratne et al., 2008).

## Peptides as Antifungal Agents

**FIGURE 3:**
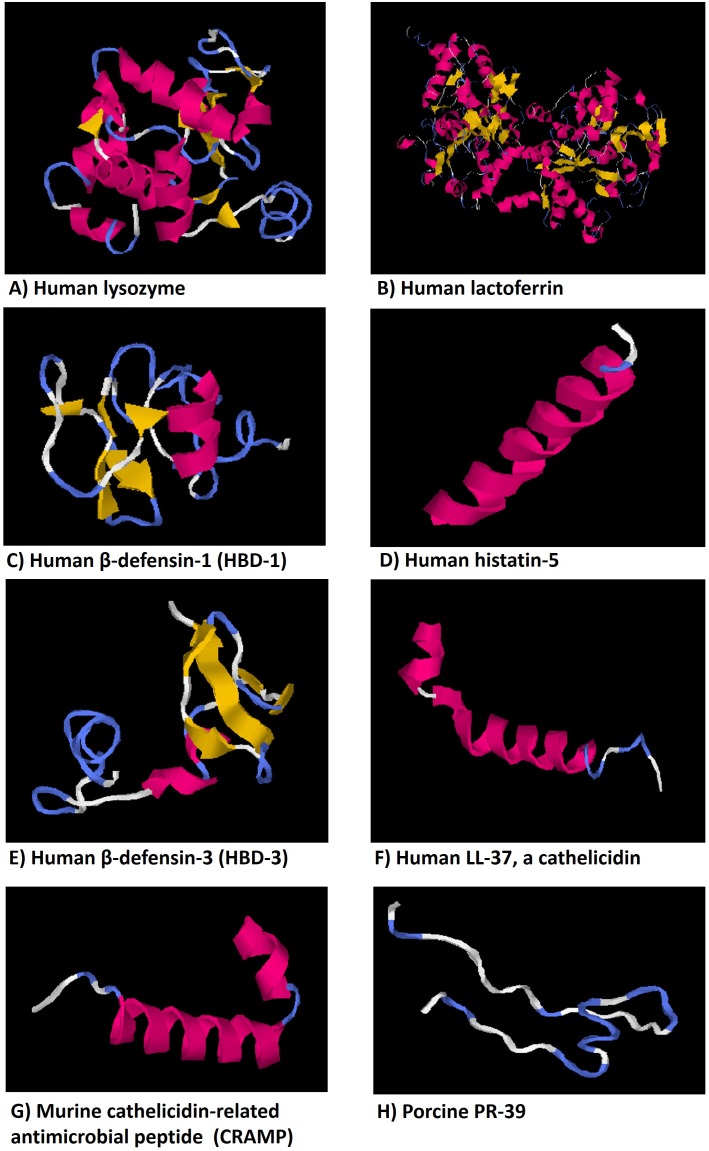
Representative models of some antifungical peptides showing alpha-helical, beta-sheet, amphiphilic structural motifs that relate forms and functions. The three-dimensional structure models of peptides were predicted using the on-line server Iterative Threading ASSEmbly Refinement (I-TASSER, Yang Zhang Lab), University of Michigan (Ann Arbor, MI, United States), from their amino acid sequences.

Some peptides isolate from various sources of body have antimicrobial properties and are also a promise in the discovery of new antimycotics. One possibility is to use molecules with antifungal properties derived from host cells to prevent or treat fungal infections (**Table 2**). There are some small cationic peptides derived from large proteins that exert antifungal activities (**Table 3**). The main mechanism of action related to these peptides is that they intensify the passage of substances through the fungal membrane favoring permeabilization. These peptides include lysozyme, lactoferrin, defensins, histatin, and cathelicidins (Mehra et al., 2012) (**Figure 3**).

**Table 2:** Alternative products with reported antifungal activities against *Candida* species showing promise for antifungal drug development.

Specific source	Biological active molecules/substances	Activity/putative mechanisms of action
New triazoles	Ravuconazole	Inhibits ergosterol biosynthesis
	Albaconazole	Inhibits ergosterol biosynthesis
	Isavuconazole	Inhibits ergosterol biosynthesis
Peptides	Lysozyme	Reduces SAP activity and secretion
	Lactoferrin	Production of cationic antimicrobial peptide lactoferricin
	Defensins	Increases membrane permeability
	Histatin	Inhibition of adhesion
	Cathelicidins	Increases membrane permeability
Plants (essential oils; terpenoids; saponins; phenolic compounds; alkaloids; peptides; proteins)	Curcumin	Inhibiting initial cell adhesion, biofilm growth, and gene expression
	*Eugenia dysenterica* (catechin derivatives and flavonoids)	Inhibits planktonic growth
	*Terminalia catappa* (hydrolysable tannins (punicalin, punicalagin), gallic acid, and flavonoid C-glycosides)	Inhibits planktonic growth
	*Carya illinoensis* (gallic acid, ellagic acid, flavonoids – rutin – and tannins – catechins and epicatechins)	Inhibits the production of germ tubes
	Quercetin, myricetin, kaempferol (flavanols)	Inhibits planktonic growth
	*Syzygium cordatum* (gallotannin)	Inhibits planktonic growth
	*Scutellaria baicalensis* (baicalein)	Induces apoptosis in *Candida albicans*
	*Ocotea odorifera* (ellagitannins)	Potent activity against *Candida parapsilosis*
	*Cymbopogon nardus* essential oil	Inhibits hyphal growth in *C. albicans*
	*Artemisia judaica* essential oil	Inhibits the formation of germination tube and biofilms in *C. albicans*
	Thymol (terpene)	Binds to ergosterol in the membrane resulting in cell death
	Carvacrol (terpene)	Alters cellular cytoplasmic membrane and induces apoptosis
	*Lannea welwitschii* (alkaloids, flavonoids, steroids, sapogenetic glycosides, tannins)	Wound healing
	*Lonicera japonica* (chlorogenic acid)	Antiwound infection, repair, and contraction

*SAP, secreted aspartic protease.*

**Table 3:** Predicted amino-acid sequences (single-letter code) of antimicrobial peptides obtained from Protein Data Bank (RCSB-PDB) or from literature reference.

Peptide	Origin	Amino-acid sequence	Accession Number (UniProtKB)	Reference
LL-37	Human	LLGDFFRKSKEKIGKEFKRIVQRIKDFLRNLVPRTES	P49913	Tomasinsig and Zanetti, 2005
CRAMP	Mouse	GLLRKGGEKIGEKLKKIGQKIKNFFQKLVPQPE	P51437	Tomasinsig and Zanetti, 2005
Lysozyme	Human	MKALIVLGLVLLSVTVQGKVFERC ELARTLKRLGMDGYRGISLANWM CLAKWESGYNTRATNYNAGDRS TDYGIFQINSRYWCNDGKTPGAV NACHLSCSALLQDNIADAVACAKRV VRDPQGIRAWVAWRNRCQNRDVRQYVQGCGV	P61626	Artymiuk and Blake, 1981
Lactoferrin	Human	MKLVFLVLLFLGALGLCLAGRRRSVQWCAVSQPEA TKCFQWQRNMRKVRGPPVSCIKRDSPIQCIQAIAE NRADAVTLDGGFIYEAGLAPYKLRPVAAEVYGTER QPRTHYYAVAVVKKGGSFQLNELQGLKSCHTGLRRT AGWNVPIGTLRPFLNWTGPPEPIEAAVARFFSASCV PGADKGQFPNLCRLCAGTGENKCAFSSQEPYFSY SGAFKCLRDGAGDVAFIRESTVFEDLSDEAERDEY ELLCPDNTRKPVDKFKDCHLARVPSHAVVARSVNG KEDAIWNLLRQAQEKFGKDKSPKFQLFGSPSGQK DLLFKDSAIGFSRVPPRIDSGLYLGSGYFTAIQNL RKSEEEVAARRARVVWCAVGEQELRKCNQWSGLSE GSVTCSSASTTEDCIALVLKGEADAMSLDGGYVYTA GKCGLVPVLAENYKSQQSSDPDPNCVDRPVEGYL AVAVVRRSDTSLTWNSVKGKKSCHTAVDRTAGWNI PMGLLFNQTGSCKFDEYFSQSCAPGSDPRSNLCA LCIGDEQGENKCVPNSNERYYGYTGAFRCLAENA GDVAFVKDVTVLQNTDGNNNEAWAKDLKLADFAL LCLDGKRKPVTEARSCHLAMAPNHAVVSRMDKVE RLKQVLLHQQAKFGRNGSDCPDKFCLFQSETKNL LFNDNTECLARLHGKTTYEKYLGPQYVAGITNLK KCSTSPLLEACEFLRK	P02788	Haridas et al., 1995
HDB-1	Human	MRTSYLLLFTLCLLLSEMASGGNFLTGLGHRSDH YNCVSSGGQCLYSACPIFTKIQGTCYRGKAKCCK	P60022	Hoover et al., 2001
HDB-2	Human	MRVLYLLFSFLFIFLMPLPGVFGGIGDPVTCLKSGAI CHPVFCPRRYKQIGTCGLPGTKCCKKP	O15263	Hoover et al., 2000
HDB-3	Human	MRIHYLLFALLFLFLVPVPGHGGIINTLQKYYCRVR GGRCAVLSCLPKEEQIGKCSTRGRKCCRRKK	P81534	Schibli et al., 2002
Porcine cathelicidin peptide PR-39	Pig	RRRPRPPYLPRPRPPPFFPPRLPPRIPPGFPPRF PPRFP-NH2	P80054	Tomasinsig and Zanetti, 2005
Histatin-5	Human	DSHAKRHHGYKRKFHEKHHSHRGY	P15516	Han et al., 2016
P318	Mouse	KIGEKLKKIAQKIKNFFAKLVAQPEQ	–	Brucker et al., 2014
HsLin06_18	Plant	FAYGGAXHYQFPSVKXFXK	–	Cools et al., 2017
HBD3-C15	Human	GKCSTRGRKCCRRKK	–	Lim et al., 2016


Accordingly, the utmost relevance in the health care field is the development of more safe and effective antimycotic agents. Therefore, this topic aimed to address new substances and/or molecules with potential antifungal activity, their clinical relevance, and mechanisms of actions. Alternative therapies with some antifungal potential include the use of synthetic agents, polymeric materials, active molecules of natural products and peptides (**Table 2**).

### New Triazoles

New triazoles antifungals are being developed and are under investigation, due to the limited number and the lack of effective antifungal. These include ravuconazole, albaconazole, and isavuconazole (**Table 2**). Preliminary studies have shown that these drugs have good pharmacokinetic profiles and low toxicity, as well as *in vitro* activity against *Candida* even in FLZ-resistant strains, with similar properties to those of FLZ and voriconazole.

Albaconazole, a broad-spectrum antifungal agent with excellent tolerability (Bartroli and Merlos, 2011), has shown great activity against *Candida* spp., both *in vitro* and *in vivo* studies, with better properties than those of FLZ. In fact, a single dose of albaconazole at a concentration almost 4× lower than that of FLZ recommended for acute vaginal candidiase has better efficacy (Pasqualotto and Denning, 2008). In addition, albaconazole showed low toxicity when administered to volunteer patients (Girmenia, 2009). Most isolates of *C. albicans* and *C. glabrata* are susceptible to treatment with albaconazole (Pasqualotto et al., 2010).

Isavuconazole is a new, second-generation triazole that has also a broad-spectrum antifungal activity. *In vitro*, isavuconazole is more active than AMB, ITC, voriconazole, 5-FC, and FLZ (MIC_50_ = 0.004, 0.5, 0.008, 0.03, 0.125, and 8 μg/ml, respectively) and has lower MIC_50_ values than those of voriconazole in the majority of *Candida* species (Pasqualotto et al., 2010). Isavuconazole has demonstrated activity against candidemia and invasive candidiasis, with an action mechanism similar to that of other triazoles and with activity in triazole-resistant fungi (Vermes et al., 2000).

Ravuconazole is structurally similar to isavuconazole and has demonstrated a good antimycotic action in FLZ resistant isolates. However, higher MIC values for ravuconazole have been reported for *Candida* isolates that display resistance to FLZ than those for susceptible isolates (Pasqualotto et al., 2010).

Lysozyme is an enzyme found in various fluids of the human body such as saliva and respiratory secretions (**Table 3** and **Figure 3**). It is classically known for its ability to kill bacteria through its muramidase activity. Further, lysozyme is active against numerous clinical isolates of *Candida* species, as well as against *Aspergillus fumigatus* and *Penicillium* species (Papini et al., 1982). However, the antifungal mechanisms of action associated with lysozyme remain subject to speculation. It is likely that lysozyme acts by reducing secreted aspartic protease (SAP, involved in *Candida* virulence) activity and secretion in *C. albicans* (Wu et al., 1999); its fungicidal activity at high concentrations likely results from damage to the cell wall or plasma membrane, causing loss of osmotic equilibrium (Wu et al., 1999).

Human lactoferrin (hLF) is a peptide that binds to iron and has protease action (**Table 3** and **Figure 3**). Lactoferrin is found in saliva and other secretions of human body. It has been found to be active against *C. albicans* and *C. krusei* (Samaranayake et al., 1997). The mechanisms of action associated with lactoferrin are likely related to production of a cationic peptide that presents a broad antimicrobial activity (Orsi, 2004). It was verified that a synthetic peptide comprising the first cationic domain of lactoferricin H (released by pepsinolysis of hLF), named hLF1-11 (**Table 3**), possesses a high antifungal activity, (Lupetti et al., 2007), beyond contributing to the clearance of infections, by stimulating the production of macrophages and dendritic cells (van der Does et al., 2012). The peptide hLF1-11 also inhibited *C*. *albicans* biofilm formation at early stages, interfering with biofilm cellular density and metabolic activity and to induce the down-regulation of biofilm and hyphal-associated genes (Morici et al., 2016).

Histatin-5 is a fragment of salivary protein histatin-3 comprising the N-terminal fragment with 24 amino acids (**Table 3** and **Figure 3**). The peptide has strong fungicidal activity, being able to kill both yeast and filamentous forms of *Candida* spp. even at low concentrations (15–30 μM); histatin-5 can also exert its fungicidal activity by binding to a candidate 67 kDa protein and then interfering with non-lytic ATP efflux (Edgerton and Koshlukova, 2000). Moreover, when histatin-5 is adsorpted to microtiter plate prior *C. albicans* biofilm formation (90 min, 24, 48, and 72 h) it is capable of reducting significantly *C. albicans* colonization interfering with biofilm formation. (Moffa et al., 2015). These data corroborate to those previously obtained by Vukosavljevic et al. (2012), who demonstrated the inhibitory effect of histatin-5 when adhered to hydroxyapatite and polymethylmethacrylate (PMMA) surfaces on *C. albicans* colonization.

Human β-defensins (HBD) are small cationic peptides that belong to the defensins family. There are three types of human β-defensin with fungicidal activity toward *C. albicans* (Krishnakumari et al., 2009; Schroeder et al., 2011; Tomalka et al., 2015) that function via the same mechanism (Krishnakumari et al., 2009), including human β-defensin-1 (HBD-1), human β-defensin-2 (HBD-2), and human β-defensin-3 (HBD-3) (**Table 3** and **Figure 3**).

Pro-inflammatory molecules such as interferon-γ, bacteria, or lipopolysaccharide can stimulate the expression of HBD-1 mRNA constitutively in endothelial tissues, by binding to toll-like receptors (TLR) (Duits et al., 2002). In addition, HBD-1 is important for control of early mucosal *Candida* infections and plays a critical role in HBD-2 expression (Tomalka et al., 2015). Cytokines, such as tumor necrosis factor, and also the contact with bacteria and fungi (Harder et al., 1997) and interleukin (IL)-1 (Sorensen et al., 2005) stimulate the expression of HBD-2 in epithelial tissue via TLR-2 (Hertz et al., 2003). HBD-3 expression is induced via binding of TGFα (transforming growth factor alpha) to its receptor EGFR (epidermal growth factor receptor). HBD-3 can be found in keratinocytes and airway epithelial cells (Sorensen et al., 2005). Of the three HBDs, HBD-3 is fungicidal against *C. albicans*, showing a minimal fungicidal concentration (MFC) of 2.5 μM; HBD-2 has a poor activity against fungi, with a MFC of 8 μM (Krishnakumari et al., 2009). Also, HBD-3 elevates Xog1 activity, an exoglucanase of *C. albicans* cell wall, resulting in reduced adherence of the yeast (Chang et al., 2012). Studies have shown that at least one mechanism of action associated with HBD-1, 2, and 3 increases the membrane permeability of *C. albicans* (Krishnakumari et al., 2009).

Other small cationic peptides include the cathelicidins [human LL-37, murine cathelicidin-related antimicrobial peptide (CRAMP), and porcine PR-39], a group of antimicrobial skin peptides produced by mast cells and by mucosal and skin epithelial cells (McCormick and Weinberg, 2000) (**Table 3** and **Figure 3**). CRAMP is both fungicidal and fungistatic against *C. albicans*, with MICs as low as 15 μM. LL-37 also has fungicidal and fungistatic activity and can be cleaved into shorter peptides with a higher fungicidal activity against *C. albicans*. Assays have shown that LL-37 and RK-31 affect membrane permeability of *Candida* cells. LL-37 also inhibits adhesion of *C. albicans* to plastics and tissues by interacting with yeast cell wall carbohydrates (Tsai et al., 2011). When the peptide LL-37 was tested for their inhibitory effects and antibiofilm properties against *C. albicans* strain, using both a crystal violet and an XTT [2,3-bis- (2-methoxy-4-nitro-5-sulfophenyl) -2H -tetrazolium-5-carboxanilide] assays showed satisfactory results, mainly in relation to the prevention of biofilms. Through violet crystal analysis, LL-37 had significant efficacy both in preventing biofilm formation and in inhibiting early formed biofilms of *C. albicans*. However, by XTT metabolic assay, LL-37 prevented biofilm formation against *C. albicans*, even at sub-minimum inhibitory concentrations (sub-MIC), but did not inhibit early biofilms (Luo et al., 2017).

Although natural proteins/peptides represent a promising therapeutic agents, they are usually extracted in small amount which makes it difficult to perform biological tests (Bondaryk et al., 2017). Stimulated by these facts, some researchers are synthetizing or modifying existing natural peptides in order to obtaining new molecules with enhanced antifungal activity and reduced toxicity. For instance, a shortened peptide variant of CRAMP, named P318 (**Table 3**), was identified by Brucker et al. (2014) and shared 67% identity with the peptide LL-37. The peptide was discovered in the islets of Langerhans of the murine pancreas. P318 showed biofilm-specific activity as it inhibited *C. albicans* biofilm formation at 0.15 μM without affecting planktonic survival at that concentration.

Mollica et al. (2017) synthesized and characterized nine new cationic peptides, rich in arginine and lysine amino acids to introduce cationic charges and in phenylalanine and leucine residues to increase lipophilicity. Four from these peptides showed a potente antifungal activity against different clinical isolates of *Candida* spp. (MIC ranged from 62.5 to 500 μg).

Cools et al. (2017) delineated and identified a linear HsLin06_18, a 19-mer peptide (**Table 3**) derived from the C-terminal part of HsAFP1, an antifungal and antibiofilm plant defensin isolated from *Heuchera sanguinea*. Synergistic combination of HsLin06_18 with caspofungin significantly reduced *in vitro* biofilm formation of *C. glabrata* and *C. albicans* on catheters, as well as *in vivo* biofilm formation of *C. albicans* strain. In addition, combination dose (4.6 μM of HsLin06_18 + 0.01 μM caspofungin) was fungicidal against planktonic cells of tested strains, killing until 80% of yeast population. Lim et al. (2016) demonstrated the antifungal and antibiofilm activities of a synthetic peptide consisting of 15 amino acids at the C-terminus of human β-defensin 3 (HBD3-C15, **Table 3**). They observed that the biofilm of *C. albicans* on dentin disks was inhibited by HBD3-C15 in a dose-dependent manner.

Despite promises, much research is still needed on hemolytic activity, instability, production modes, interaction with high salt concentrations, anti-virulence activity, and poor ability to cross physiological barriers of these peptides, which could limit their use in the clinic.

### Plants as a Source for Anti-*Candida* Natural Compounds

The use of plants and their bioactive molecules in the treatment of candidiasis has emerged as a promising alternative to traditional drugs against resistance which has developed in the *Candida* genus. Antifungal substances derived from plants can selectively act on different targets with fewer side effects. In addition, the practice of phytotherapy is inexpensive; therefore, floral diversity has resulted in an increase in potential usage in populations experiencing economic difficulties. This review comments on some of the extracts of plants or their metabolites that *in vitro* and *in vivo* studies have already demonstrated a potential antifungal activity.

There are some families of plants that are more studied than others as the *Combretaceae* and *Acanthaceae*. Studies have shown that leaves, seeds, fruits, and flowers have the most enriched plant components. Leaves, as well as the seeds and fruits of plants have higher levels of phenolic compounds. The concentration of these compounds also depends on the nature of the chemical used as solvent in the extraction process as well as on the growth and storage conditions (Martins et al., 2015a). The most used solvents in extraction processes are dichloromethane, methanol, ethanol, ethyl acetate and n-butanol. (Martins et al., 2015a).

Recently, some authors have verified and evaluated the biological activity of plant products against *Candida* species. *Lonicera japonica*, a medicinal plant of folk medicine of China used to treat some diseases, was investigated by Chen et al. (2012) for the *in vivo* activity of an ethanol extract of its aerial parts. The extract showed a very strong antimicrobial activity against *C. albicans* and *C. tropicalis* and potent wound healing capacity; further, enhanced production of anti-inflammatory cytokines was observed. In this way, the authors suggested that both activities detected in this extracts act synergistically accelerating the process of wound healing.

Some properties of *Lannea welwitschii* and *Justicia flava* were investigated by Agyare et al. (2013). Methanolic extract of *Lannea welwitschii* leaves was antimicrobial against clinical strains of *C. albicans* and other microorganisms. The MIC for *C. albicans* was 2.5 mg/mL. Treatment with an extract from both plants resulted in a significant decrease in wound size and increase in wound tensile strength. A preliminary phytochemical screening of extracts revealed tannins, flavonoids, alkaloids, and glycosides as compounds. These results corroborate the use of these vegetable extracts in treatment of wounds and infections in phytotherapy.

Pereira et al. (2014) studied the activity of *Pyrostegia venusta* crude flower extracts, fractions, and pure compounds against isolates of *Candida* spp. and showed an effective broad spectrum antifungal activity. Nordin et al. (2014) reported anticandidal activity in an extract of *Piper betle* leaves; in fact, the extract inhibited the growth of all *Candida* species tested. Isa et al. (2014) also verified antimicrobial property of four different extracts of *Strychnos spinosa* and their fractions against American Type Culture Collection (ATCC) strains of *C. albicans* and *C. albicans* isolates (MICs of 0.16 and 0.63 mg/mL, respectively). Otari et al. (2014) described that silver nanoparticles containing *Manilkara zapota* seed extracts showed good activity against *Candida* species.

Shahzad et al. (2014) evaluated the antifungal potential of 14 polyphenols against various *C. albicans* clinical isolates in terms of planktonic and sessile MICs (PMICs and SMICs, respectively). Among these, 7 were able to inhibit planktonic growth. The most effective was pyrogallol (PMIC_50_ = 78 μg/mL) and curcumin (PMIC_50_ = 100 μg/mL). In addition, curcumin inhibited adhesion capability of cells and demonstrated anti-biofilm activity against *C. albicans* (SMIC_50_ = 50 μg/mL).

Martins et al. (2015b) evaluated ten different plant extracts commonly used in folk medicine for antifungal activity against *Candida* spp. They verified that hydro-methanolic extracts of leaves from two of these plants, *Juglans regia* and *Eucalyptus globulus*, demonstrated excellent antimycotic property against all *Candida* strains. Goncalves et al. (2015) described anticandidal activity in a *Cynomorium coccineum* methanol extract, which showed excellent action against *C. guilliermondii* and *C. krusei*, showing very low MIC values (0.025 mg/mL). Moraes et al. (2015) investigated the antimycotic property of a hydroethanolic extract of *Uncaria tomentosa* and some of its fractions against resistant *Candida* spp. and verified that the water-insoluble fraction showed significant antifungal activity.

Akroum (2017) showed antifungal activity in an acetylic extract of *Vicia faba* against *C. albicans* (MIC of 0.010 mg/mL) *in vitro*. Further, mortality rates were reduced in mice that were administered with the extract (20 μg/mL) for treatment of candidiasis.

Correia et al. (2016) evaluated the antifungal properties of six plants from Brazilian Cerrado commonly used in folk medicine (ethanolic and aqueous extracts) against different *Candida* reference strains using the disk diffusion method and determining MICs. Among these plants, the most promising were *Eugenia dysenterica* and *Pouteria ramiflora.* They showed excellent activity against *C. tropicalis*, *C. famata*, *C. krusei*, *C. guilliermondii*, and *C. parapsilosis* with low MICs values. A phytochemical screening of active extracts from these plants disclosed as main components flavonoids and catechins.

Terças A.G. et al. (2017) found antifungal properties in crude extract and fractions (n-butanolic and ethyl acetate ones) from *Terminalia catappa* leaves via the agar diffusion and microdilution tests when analyzed against *Candida* spp.; hydrolysable tannins (punicalin, punicalagin), gallic acid, and flavonoid *C*-glycosides are likely the active components. Todorovic et al. (2017) verified the antifungal activity of polyphenols (flavanol monomers such as epicatechin and catechin, and procyanidin oligomers) of alkalized/non-alkalized *Theobroma cacao* powders against *C. albicans* (ATCC 10231) and determined a MIC value of 5.0 mg/mL using the broth microdilution method.

Phytochemicals present in leaves of *Carya illinoensis* were first identified by Bottari et al. (2017), and the antimicrobial activity of their aqueous and ethanolic extracts was determined. Both extracts had MIC values against seven *Candida* reference strains between 25 mg/mL and 6.25 mg/mL. Phenolic acids (gallic acid and ellagic acid), flavonoids (rutin), and tannins (catechins and epicatechins) were likely responsible, in part, for the activity against *Candida* strains. Further, the extracts inhibited the production of *C. albicans* germ tubes.

Important biologically active molecules are found in plants (Martins et al., 2015c). Polyphenols are a kind of substance most found in plants; they are low molecular weight naturally occurring organic compounds that contain one or more phenolic groups (Daglia, 2012; Shahzad et al., 2014). Further, polyphenols perform various substantial functions in plant physiology and, therefore, can be found, in lesser or greater quantity, in all of them. Phenolic acids, flavonoids, tannins, coumarins, are some examples of phenolic compounds found in and extracted from medicinal plants (Daglia, 2012). Research has shown that polyphenols have potentially healthy effects in humans, working primarily as anticancer, antihypertensive, anti-allergen, anti-inflammatory, antioxidants, and antimicrobial agents. The antimicrobial activity of polyphenols has been extensively investigated mainly against bacteria (Daglia, 2012).

Nevertheless, the antifungal activity of some of the above-mentioned phenolic compounds remains unknown and determining the antifungal activity of such compounds remains an open area of research. Reports of studies of phenolic compounds against *Candida* are still scarce. There are few studies on the mechanism of action of the substance, cytotoxicity, the synergism with traditional antifungals drugs and their anti-virulence activities (such as inhibition of biofilm formation, interference of adhesion capability, interference of hyphal formation or inhibition of exoenzymes production).

Flavan-3-ols, flavonols, and tannins have received the most attention among the known polyphenols, attributable to their large spectrum of efficacy and high antimicrobial property, which have been shown in many bacteria strains and isolates. Further, virulence factors of bacteria may be influenced by polyphenols and also these substances can act in synergism with antibiotics; consequently, those polyphenols are also the most studied in relation to their anti-*Candida* activities. Thus, we reviewed the antifungal activities related in scientific literature of the polyphenols considered most actives and their mechanisms of action.

Flavonoids are a class of natural compounds with several known protective activities, including antifungal activity. The flavonoids include subclasses such as chalcones, flavones, isoflavones, flavonols, flavanols (flavan-3-ol), and anthocyanidins (Seleem et al., 2017). The activity of flavonols such as quercetin, myricetin, and kaempferol has been described in *C. albicans*. Herrera et al. (2010) showed inhibition of *C. albicans* growth with isolated flavonols from propolis using quercetin in an agar microdilution method, obtained a variation of MIC values from 197 to 441 μg/mL. These same authors found similar results for myricetin and kaempferol against *Candida* species (Herrera et al., 2010). Other studies have reported antimicrobial activity (Avila et al., 2008; Batovska et al., 2009) for other propolis polyphenols such as flavanone (pinocembrin and pinostrobin) and chalcones (2,4-dihydroxychalcone and 2,4-dihydroxy-3-methoxychalcone). The flavanols subclass (flavan-3-ol) and gallotannin, extracted from *Syzygium cordatum*, also showed inhibitory properties on the growth of *C. albicans*, with a MIC of 0.195 mg/mL in a microdilution test (Mulaudzi et al., 2012). Serpa et al. (2012) isolated baicalein, belonging to a subclass of flavones, from *Scutellaria baicalensis*, and induced apoptosis in *C. albicans*, with a MIC value of 26 μg/mL. Apigenin, a flavone isolated from propolis, had a MIC of 441 μg/mL against *C. albicans*, as shown using a microdilution test.

Tannins are polyphenolic compounds present in various plant parts, such as the roots, flowers, leaves, fruits, and seeds. Tannins are divided into ellagitannins (hydrolysable tannins), proanthocyanidins (condensed tannins) and gallotannins (Duval and Avérous, 2016). They have the ability to interact with and precipitate macromolecules such as proteins (dos Santos et al., 2017), as well as having antimicrobial properties. However, the mechanisms underlying the antimicrobial action of tannins in different microorganisms such as bacteria and fungi are still under investigation (Morey et al., 2016; Xu et al., 2016; dos Santos et al., 2017).

The knowledge that wood durability could be attributed to the accumulation of ellagitannins, with the ability to precipitate proteins and/or withdraw metallic cofactors acting as a microbial barrier, raised the suspicion that ellagitannins would have antifungal activity. Accordingly, ellagitannins isolated from *Ocotea odorifera*, a plant commonly used in Brazil in folk medicine, have a very good activity against *C. parapsilosis* (Yamaguchi et al., 2011). dos Santos et al. (2017) verified that encapsulated tannins from *Acacia mearnsii* have moderate activity against the filamentous fungi *Aspergillus niger* (ATCC 9642) and *C. albicans* (ATCC 34147).

Other phytosubstances reported to be active against yeasts and fungi include essential oils, which are derived from aromatic medicinal plants (Reichling et al., 2009; Shahid et al., 2009; Centeno et al., 2010; Vale-Silva et al., 2010; Pitman et al., 2011; Sienkiewicz et al., 2011). Essential oils are rich in monoterpenes, sesquiterpenes, and/or phenylpropanoids, considerate volatile organic compounds. Essential oils are present in various plant species. Mondello et al. (2003) proposed that tea tree oil could be used in antifungal therapy, because it showed efficacy against multidrug-resistant *Candida* species *in vitro* and against mucosal candidiasis *in vivo*; they further documented that terpinen-4-ol was the main substance to contribute to the anticandidal activity.

Several oils have demonstrated activity against *Candida* species. Essential oils from *Carica papaya* have inhibitory effects against *Candida* species, as shown via the agar diffusion method and the microdilution method, with MICs between 4 and 16 mg/mL and MFCs between 16 and 64 μg/mL (Sharma H. et al., 2016; Sharma Y. et al., 2016; He et al., 2017). Minooeianhaghighi et al. (2017) tested a combination of essential oils from *Cuminum cyminum* and *Lavandula binaludensis* against *C. albicans* isolates, showing growth inhibition at concentrations between 3.90 and 11.71 μg/mL. Essential oils from *Cymbopogon nardus* have also shown antimicrobial potential against *Candida* species, with MICs between 250 and 1000 μg/mL and with inhibition of hyphal growth in *C. albicans* at concentrations between 15.8 and 1000 μg/mL (De Toledo et al., 2016). In addition to inhibiting biofilm formation (Abu-Darwish et al., 2016), essential oils from *Artemisia judaica* have been shown to inhibit the formation of germination tubes in *C. albicans*, with 80% inhibition of filamentation at a concentration of 0.16 μL/mL. Köse et al. (2016) demonstrated the fungicidal potential of essential oils from *Centaurea baseri* against *Candida* species, with a MIC of 60 μg/mL.

Sharifzadeh et al. (2015) observed that essential oils from *Trachyspermum ammi* have anticandidal effects against isolates of *C. albicans*, some of which were resistant to FLZ. Gavanji et al. (2015) have compared the efficiency of herbal essences from *Foeniculum vulgare, Satureja hortensis, C. cyminum*, and *Zataria multiflora* against *C. albicans.* Essential oils from *Z. multiflora* showed the best anticandidal activity of those tested, with MIC and MFC values of 34 and 64 μg/mL, respectively.

Among monoterpenes there is thymol (2-isopropyl-5-methylphenol), (Sánchez et al., 2004). It is the most abundant constituent in essential oils from *Thymus vulgaris* (thyme) (De Lira Mota et al., 2012) and the major component of essential oils from *Origanum vulgare* (oregano) (Romero et al., 2012). Antifungal activity of thymol was investigated by de Castro et al. (2015) against *Candida* strains. They verified fungistatic and fungicidal activity, mechanisms of action and mode of interactions in combination with nystatin. Thymol had antifungal properties, with MIC of 39 μg/mL against *C. albicans* and *C. krusei*, and MIC of 78 μg/mL against *C. tropicalis*. Antifungal assays also showed an eightfold increase (from 39.0 to 312.5 μg/mL) in thymol MIC values against *C. albicans* in the presence of exogenous ergosterol, indicating that thymol binds to ergosterol in the plasma membrane, thereby increasing ion permeability and resulting in cell death. Combination of thymol and nystatin resulted in synergy.

Terpenoids exhibit a very good antimycotic activity against blastopores and filamentous forms growth of *C. albicans* at concentrations that are non-toxic to HeLa cells (Zore et al., 2011). Accordingly, terpenoids may be useful as a future antifungal chemotherapeutic agent, in addition to its synergistic effects with conventional drugs such as FLZ (Zore et al., 2011). Further, in experiments realized by Fan et al. (2011), rubiarbonol G, a triterpenoid from *Rubia yunnanensis*, showed potent antimicrobial activity against *C. albicans*, with a MIC of 10.5 μg/mL.

The anti-biofilm activity of terpenes, along with the efficacy of thymol, geraniol, and carvacrol in the treatment of *Candida* infections associated with the use of hospital devices has been related (Dalleau et al., 2008). Mechanisms underlying the effects associated with carvacrol include alterations of the cellular cytoplasmic membrane and induction of apoptosis, as shown in an *in vitro* macrodilution study in *Candida* species (Mulaudzi et al., 2012).

Phenylpropanoids are other naturally occurring compounds frequently studied for their anti-*Candida* properties; they are categorized as coumarins, phenylpropanoic acid, and lignans (Lu et al., 2017). Navarro-García et al. (2011) and Raut et al. (2014), found that a coumarin (scopoletin) and two phenylpropanoic acids (salicylaldehyde and anisyl alcohol) have antifungal property against *C. albicans*, with MICs of 25, 31, and 31 μg/mL, respectively.

Taken together these data show that plants contain molecules possessing high bioactive potential. However, the process of discovering bioactive molecules is complex and time-consuming, involving the isolation, identification, and optimization of pharmacokinetic and pharmacodynamic properties, as well as the selection of lead compounds for further drug development.

### Synergistic Effects of Plant Extracts or Their Phytoconstituents With Traditional Agents

The knowledge about synergistic effects of plant extracts or their phytoconstituents with traditional agents is nowadays a type of study that is indispensable, since some screening assays, most realized *in vitro*, have evidenced that plant extracts are less effective than existing antifungal agents (Newman and Cragg, 2012) and that extracts with MIC of 1000 μg/mL are considered inefficient (Morales et al., 2008).

The use of drug combinations in treatment of infections by fungi is a preferred strategy clinically. Although combination of medications requires a careful evaluation of the synergistic, antagonistic, and agonist properties of the drugs involved (Lewis and Kontoyiannis, 2001), it has been used successfully in many cases of fungal infection (Serena et al., 2005; Espinel-Ingroff, 2009; Bink et al., 2011; Rodrigues et al., 2014) (**Table 1**). The synergistic effects of drugs are primarily attributable to cell wall damage as one antifungal component potentiates the activity of drugs exactly against some constituent of plasma membrane. Alternatively, a compromised cell wall has increased permeability and could facilitate movement of drugs across the cell membrane to their targets.

Accordingly, the synergistic effect of plant extracts or their biomolecules in combination with conventional antimicrobial agents (or with some other different extract or biosubstance) against clinical multidrug-resistant microorganisms represents a successful therapeutic approach (Mukherjee et al., 2005). Advantages of combination therapies include lower doses of antifungal agent, possible synergistic actions between antifungals, and less development of drug resistance. The objective of this strategy is to maximize the antifungal effects. Some examples are addressed herein.

Tangarife-Castaño et al. (2011) reported synergy between essential oils or plant extracts associated with antifungal drugs when used as anti-*C. albicans* agents. The best synergistic effects were obtained for combination between itraconazole and *P. bredemeyeri* extract [fractional inhibitory concentration index (FICI) range of 0.09–0.13] against *C. albicans.*

Chanda et al. (2013) verified a synergistic potential when methanolic extract of *T. catappa* leaves was combined with nystatin or AMB against *C. albicans* (ATCC 209), *C. neoformans* (National Collection of Industrial Microorganisms [NCIM] 3542), *C. glabrata* (NCIM 3448), *C. apicola* (NCIM 3367), and *Trichosporon beigelii* (NCIM 3404). As such, maximum synergy was observed against *C. apicola*.

Santos et al. (2013) related the antimycotic properties of an ethanol extract of *Hyptis martiusii* (EEHM) against *C. albicans, C. krusei*, and *C. tropicalis.* They verified synergistic antifungal activity for EEHM in combination with metronidazole when used against *C. tropicalis*.

Avijgan et al. (2014) reported a synergistic effect between an *Echinophora platyloba* ethanolic extract and different azoles against isolates of *C. albicans* from vaginal secretions of patients with recurrent vulvovaginitis. MIC and MFC values ranged from 3.1 to 6.25 mg/mL and 6.2 to 12.5 mg/mL, respectively, showing potent synergistic effects of the *E. platyloba* ethanol extract in combination with itraconazole and FLZ.

Combination between thymol and nystatin was found to have synergistic effects against *Candida* species (de Castro et al., 2015), reducing the MICs of both products by 87.4% and generating a fractional inhibitory concentration (FIC) index of 0.25.

Synergism between a water insoluble fraction (WIF) from *U. tomentosa* (cat’s claw) bark and the agents terbinafine or FLZ was investigated against seven resistant isolates of *C. glabrata* and *C. krusei* via the checkerboard procedure using a microdilution technique (Moraes et al., 2015). Synergism was observed between the *U. tomentosa* WIF and terbinafine, as well as between the *U. tomentosa* WIF and FLZ. The most efficacious synergistic effects leading to cell damage were unequivocally attributed to a combination of the *U. tomentosa* WIF and terbinafine (1.95:4.0, l g/mL), as well as the *U. tomentosa* WIF and FLZ (1.95:8.0, l g/mL). Moraes et al. (2015) also demonstrated, through differential scanning calorimetry and infrared analysis, that intermolecular interactions between the *U. tomentosa* WIF components and either terbinafine or FLZ occurring outside the cell wall are likely responsible for synergistic effects observed between substance. An action on constituents of the cell wall was suggested, independent of ABC efflux pump mechanisms.

Ngouana et al. (2015) conducted a bioguided screening with sub-fraction combinations of *T. catappa, Terminalia mantaly*, and *Monodora tenuifolia* against *C. albicans*, *C. glabrata*, *C. parapsilosis*, and *C. neoformans* isolates, as well as the *C. albicans* NR-29450 reference strain. They observed synergistic interactions between subfractions combinations. A combination of *M. tenuifolia* and *T. mantaly* (C36/C12) sub-fractions showed synergistic interactions and fungicidal effects against most tested strains.

Cavalcanti Filho et al. (2017) verified that the methanolic extract of *Buchenavia tetraphylla* is a great source of antimicrobial compounds that enhance the action of FLZ against different *C. albicans* isolates from vaginal secretions as well as azole-resistant isolates. The extract increased the action of FLZ in most strains through additive (20% of strains) or synergistic (60% of strains) effects.

Although many *in vitro* studies examining synergistic effects among potential antifungal biomolecules and traditional antifungal agents have been reported in the literature as described herein, the mechanisms underlying these synergistic effects are poorly understood. Several randomized and controlled analyzes have been performed with the objective of verifying the efficacy and risks of using traditional antifungal combinations; however, the high cost, reduced number of clinical cases and existence of confusing variables have resulted in contradictory and poor results. Therefore, it is extremely relevant the constant search for new phytocompounds to examine carefully possible synergism between them and conventional antifungal agents in order to obtain more insight. A lack of consensus in the medical clinical emphasizes the need to conduct further clinical trials using combinations of antifungals. The experiments and results addressed herein support further investigation of new plant constituents with antifungal properties and the efficacy of combination therapies involving phytocomponents and traditional antifungal agents as an important start for the development of unusual and original antifungal therapies.

## Concluding Remarks

*Candida* species are highly resistant to existing antifungal agents and can adapt to different host niches thus representing a serious risk to human health. The mechanisms underlying development of antifungal resistance are complex and involve multiple pathways and genes. Further, these mechanisms continue to change and evolve, challenging the medical clinic and exacerbating the need for discovering original therapies against *Candida* diseases. In this way, identification of new bioactive compounds as well as development of original formulations of antifungals and combinations involving active biomolecules and conventional agents represents the possibility for a successful therapeutic approach.

## Author Contributions

CAM, CV, GOS, FR, and AL conceived and designed the review. CAM, CV, GOS, FR, AL, MSC, AF, FN, RR, EP, and MA wrote the paper. All authors read and approved the final manuscript.

## Conflict of Interest Statement

The authors declare that the research was conducted in the absence of any commercial or financial relationships that could be construed as a potential conflict of interest.
